# Disgust Propensity, Not Disgust Sensitivity, Shapes the Reactivity of a Subjective Disgust Circuit in Humans

**DOI:** 10.1002/hbm.70577

**Published:** 2026-06-04

**Authors:** Xianyang Gan, Zihao Zheng, Ran Zhang, Feng Zhou, Ting Xu, Nan Qiu, Junjie Wang, Heng Jiang, Shan Gao, Yu Wu, Benjamin Klugah‐Brown, Dezhong Yao, Benjamin Becker

**Affiliations:** ^1^ The Clinical Hospital of Chengdu Brain Science Institute, MOE Key Lab for Neuroinformation, School of Life Science and Technology University of Electronic Science and Technology of China Chengdu China; ^2^ China‐Cuba Belt and Road Joint Laboratory on Neurotechnology and Brain‐Apparatus Communication University of Electronic Science and Technology of China Chengdu China; ^3^ Faculty of Psychology Southwest University Chongqing China; ^4^ Key Laboratory of Cognition and Personality Ministry of Education Chongqing China; ^5^ School of Foreign Languages, University of Electronic Science and Technology of China Chengdu China; ^6^ MIND & AI Lab, Department of Psychology The University of Hong Kong Hong Kong Special Administrative Region China; ^7^ SRT AI, Society & Social Dynamics, Faculty of Social Sciences The University of Hong Kong Hong Kong Special Administrative Region China

**Keywords:** disgust, emotion, fMRI, insula, propensity, sensitivity, striatum

## Abstract

Disgust constitutes an evolutionary adaptive defensive‐avoidance response, yet humans vary markedly in their dispositional tendency to experience disgust (disgust propensity) and in their negative appraisal of such experience (disgust sensitivity). Conceptual frameworks and neuroimaging studies suggest that these traits may differentially modulate neural responses to disgust‐eliciting stimuli; however, methodological constraints have left their precise roles unresolved. Our comparably large fMRI study (*n* = 142) therefore aimed to systematically determine how trait disgust modulates neural responses to carefully selected and validated disgust‐specific visual stimuli across varying levels of subjective disgust experience. The whole‐brain voxel‐wise regression analyses revealed a differential pattern of neural associations between the two disgust traits, with disgust propensity, but not disgust sensitivity, modulating disgust‐related neural activity in the anterior, middle, and posterior insula, as well as the caudate, putamen, thalamus, hippocampus, and parahippocampal gyrus. Mediation and network‐level analyses further supported this partly dissociable pattern by showing that disgust propensity shapes disgust experience via insula‐striatal‐hippocampal pathways. Together, these findings provide evidence for a differential association between disgust propensity and disgust sensitivity with disgust‐related neural responses and elucidate how trait disgust shapes subjective experiences. They further suggest that disgust propensity and the identified systems may represent promising targets for the regulation of disgust‐related pathology.

## Graphical abstract

Using task‐based fMRI with online subjective disgust ratings and trait‐level data from a comparably large study, we found a differential pattern of neural associations between two disgust traits: disgust propensity, not disgust sensitivity, modulated neurofunctional activity toward disgust elicitors and mediated the link between subjective disgust experience and activity in the disgust circuit.

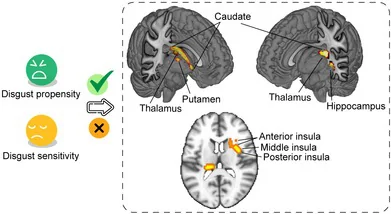

## 1 Introduction

Disgust has been conceptualized as an evolutionary adaptive defensive‐avoidance response, which may have initially evolved in mammalian rejection of potentially poisonous food (Chapman et al. 2009; Rozin and Fallon 1987; Rozin et al. 2009; Tybur et al. 2013). Consistent with its proposed evolutionary defensive‐avoidance function, the feeling of disgust is primarily elicited by poisonous as well as contaminated and contagious stimuli, indicating a broader function in pathogen avoidance (Gan et al. 2024, 2022; Jones 2007; Vicario et al. 2017). However, despite this well‐established functional account, a comprehensive understanding of the neural mechanisms underlying disgust remains incomplete.

Over the past three decades, numerous studies have used functional magnetic resonance imaging (fMRI) to examine the neural substrates of disgust in humans. The majority of studies in this field typically present participants with disgust‐eliciting stimuli from different sensory modalities and average the participants' neural responses on the group level. These studies enable us to identify the core brain systems involved in disgust processing, with recent large‐scale meta‐analytic syntheses showing that disgust engages the defensive‐avoidance circuit, including the insula, amygdala (extending into the parahippocampal gyrus), basal ganglia, as well as prefrontal, parietal, and temporo‐occipital regions (Gan et al. 2025, 2022).

However, these studies did not take into account individual differences in trait disgust, such as disgust propensity—the general tendency to respond with disgust in a certain situation—or disgust sensitivity, referring to how unpleasant the experience of disgust is appraised (van Overveld et al. 2010, 2006). Individuals with greater disgust propensity are more likely to perceive stimuli as disgust‐eliciting (van Overveld et al. 2010), which may result in stronger activation of disgust‐related brain regions compared to individuals with lower levels of disgust propensity (Calder et al. 2007). Elevated levels of disgust propensity have been linked to behavioral avoidance (de Jong and Muris 2002; Deacon and Olatunji 2007; Goetz et al. 2013; van Overveld et al. 2010), and together, these factors have been implicated in the etiology of various psychopathological conditions (e.g., eating disorders and, in particular, different forms of anxiety disorders; see Aharoni and Hertz 2012; Moretz and McKay 2008; Olatunji et al. 2017; Olatunji et al. 2010; Olatunji and Kim 2024; van Overveld et al. 2011). However, in the development of psychopathology, what matters may not only involve disgust propensity but also disgust sensitivity (van Overveld et al. 2010, 2006). Notably, both types of trait disgust have been shown to be differentially associated with symptoms of anxiety disorders (Davey 2011; Goetz et al. 2013; Olatunji et al. 2011, 2009; van Overveld et al. 2010, 2006). Combined, these findings underscore the relevance of trait‐level individual differences in disgust, yet it remains unclear whether and how disgust propensity and disgust sensitivity differentially shape neural responses to disgust‐inducing stimuli.

In response to this need, another line of research has utilized correlational approaches (e.g., regression analyses) to investigate how individual differences in trait disgust modulate neural responses during disgust induction. However, findings remained highly inconsistent. The insula has been widely recognized as a key region associated with disgust propensity (Olatunji et al. 2017; Vicario et al. 2017). Some studies observed an association between trait disgust propensity and activation of the insula to disgust‐eliciting stimuli (Calder et al. 2007; Mataix‐Cols et al. 2008), while other studies failed to observe such results (Caseras et al. 2007; Schienle et al. 2005; Stark et al. 2005). Furthermore, three early studies compared the modulatory roles of both disgust traits (propensity and sensitivity) and yielded conflicting findings. Two of them revealed an association between disgust sensitivity, rather than disgust propensity, and insula activity in response to disgust stimuli (Borg et al. 2012; Watkins et al. 2016), whereas the third study found the opposite result—that disgust propensity, not sensitivity, modulated insula activity (Schäfer et al. 2009). These discrepancies may be due to experimental and technical limitations of the previous studies. First, none of the studies collected participants' momentary subjective disgust experience evoked by each disgust stimulus while undergoing fMRI (also termed as “state disgust”), but instead employed the categorical approach of comparing a priori assigned high disgust versus neutral stimuli. The neglect of incorporating individual differences in state disgust into the statistical model may inherently capture neural activity related to salience, arousal, or autonomous reactivity, thereby obscuring the correspondence between subjective feelings and their brain representation (Ferraro et al. 2022; Gan et al. 2024; Pujol et al. 2018; Stark et al. 2007; Zhang, Gan, et al. 2025). Second, the majority of visual stimuli used to induce disgust experience in most of the studies were selected from standardized affective picture databases (e.g., International Affective Picture System, IAPS; Lang et al. 2008) that were not specifically designed to induce disgust and may also induce high levels of general negative affect (Mataix‐Cols et al. 2008), a response that is distinguishable from subjective disgust experience on the neural level (Čeko et al. 2022; Gan et al. 2024). Third, differences in the operationalization of trait disgust across studies may have contributed to the divergent findings. Specifically, some studies (Calder et al. 2007; Caseras et al. 2007; Mataix‐Cols et al. 2008) used the Disgust Scale (Haidt et al. 1994), and others (Schäfer et al. 2009; Schienle et al. 2005; Stark et al. 2005) used the Questionnaire for the Assessment of Disgust Proneness (Schienle et al. 2002), both of which primarily assess disgust propensity but do not explicitly distinguish between propensity and sensitivity. In contrast, studies reporting associations with disgust sensitivity (Borg et al. 2012; Watkins et al. 2016) employed the Disgust Propensity and Sensitivity Scale‐Revised (DPSS‐R), which is the first scale specifically designed to differentiate between these two components (van Overveld et al. 2006). Notably, unlike the Disgust Scale (Haidt et al. 1994) or the Questionnaire for the Assessment of Disgust Proneness (Schienle et al. 2002), the DPSS‐R does not contain specific disgust elicitors, which is vital to prevent spurious associations between trait disgust scores and actual state disgust responding during the experiment owing to content overlap of stimuli (Borg et al. 2012; Watkins et al. 2016). Such differences in construct operationalization may lead to distinct and potentially non‐comparable patterns of associations with neural responses. Finally, all studies—except for Mataix‐Cols et al. (2008)–examining associations between trait disgust and neural reactivity focused on a priori defined brain regions of interest (ROI) analyses and did not utilize a hypothesis‐free and more conservative voxel‐wise whole‐brain correlation approach.

The present study thus seeks to determine how individual differences in both disgust propensity and disgust sensitivity influence neural responses to a diverse set of validated disgust‐specific affective pictures (from the DIsgust‐RelaTed‐Images (DIRTI) disgust images database, see Haberkamp et al. 2017; for details, see Methods) in a large sample of healthy individuals (*n* = 142). Importantly, the included pictures span a wide range of disgust intensities and are expected to evoke varying levels of subjective disgust experience, assessed online during fMRI (details see Gan et al. 2024). Here, we chose the DPSS‐R over content‐dependent trait disgust measures such as the Disgust Scale (Haidt et al. 1994) or the Questionnaire for the Assessment of Disgust Proneness (Schienle et al. 2002) because it enables a clear conceptual distinction between disgust propensity and disgust sensitivity, which is central to the aims of the present study. Briefly, we utilized the behavioral and neural data to determine the modulatory role of trait disgust on neural responses under varying levels of state disgust, as captured by multiple contrast‐based comparisons (e.g., high disgust > neutral, high disgust > moderate disgust), using a whole‐brain voxel regression approach. We subsequently employed mediation and network‐level analyses to link trait variations with subjective experience and neural responses. Specifically, we conducted a series of mediation analyses to further examine the relationship between state disgust, brain activation, and trait disgust. In addition, network‐level analyses were performed by integrating meta‐analytic connectivity mapping (MACM) and resting‐state functional connectivity (RSFC) to delineate the connectivity profiles of brain regions identified through the core regression analyses. These systematic analyses enable us to achieve a more comprehensive characterization of how trait disgust shapes neural responses to disgust‐inducing stimuli.

Based on prior theoretical and empirical work, we hypothesized that disgust propensity would be positively associated with neural responses in disgust‐related regions (e.g., the insula and subcortical structures), given its role in determining the likelihood of experiencing disgust in response to external stimuli. In contrast, due to the lack of substantial and convergent neurofunctional findings in this domain, we did not formulate a strong a priori hypothesis regarding disgust sensitivity, as it primarily reflects the negative appraisal of the disgust experience rather than the propensity to generate such responses. Importantly, the present design may facilitate disentangling these potential contributions. By incorporating trial‐by‐trial state disgust ratings, we are able to model individual differences in subjective experience directly, rather than relying solely on categorical stimulus contrasts. Moreover, the use of validated disgust‐specific images spanning a wide range of intensity allows for a more fine‐grained characterization of neural responses across different levels of disgust. Together, these features enhance the sensitivity of the present design to detect and dissociate the respective contributions of disgust propensity and disgust sensitivity to neural responses across varying levels of state disgust.

## 2 Methods

### 2.1 Participants

A total of 146 healthy, right‐handed participants with normal or corrected‐to‐normal vision were initially recruited. Exclusion criteria included MRI contraindications, neurological or psychiatric disorders, medication or substance use. Four participants were excluded for head motion > 3 mm, resulting in a final sample of 142 (75 females; mean ± s.d. age 22.23 ± 2.60 years). The study was part of a larger project (details see Gan et al. 2024) and was approved by the Ethics Board of the University of Electronic Science and Technology of China. Written informed consent was obtained from each participant before the experiment.

### 2.2 Self‐Report Trait Disgust Questionnaire

The DPSS‐R contains 16 items constituting two validated trait disgust subscales: an 8‐item disgust propensity subscale and an 8‐item disgust sensitivity subscale. The translation of the DPSS‐R into Chinese adhered to a strict forward and backward translation procedure by bilingual English professors from the school of foreign languages and psychological professionals. The final iteration yielded a scale assumed to accurately capture the context‐adjusted English items of DPSS‐R.

Prior to scanning, participants completed the Chinese DPSS‐R (see Supporting Information), rating each statement on a 5‐point Likert scale (1 = never, 5 = always). Example items include “Disgusting things make my stomach turn” (propensity) and “I worry that I might swallow a disgusting thing” (sensitivity). In our sample, the Cronbach's *α* coefficient for the disgust propensity subscale was 0.80, and the Cronbach's *α* coefficient for the disgust sensitivity subscale was 0.77, both of which were highly similar to the original English version of DPSS‐R (i.e., Cronbach's *α* = 0.78 for the disgust propensity subscale, and Cronbach's *α* = 0.77 for the disgust sensitivity subscale; for more details see van Overveld et al. 2006), indicating a reliable Chinese version of DPSS‐R.

### 2.3 fMRI Task

The present study encompassed two disgust fMRI experiments with (partly) different stimuli that were originally designed as a discovery‐replication design (see Gan et al. 2024). Specifically, the dataset was originally collected to evaluate the predictive performance of the visually induced disgust signature across independent cohorts, and participants were therefore recruited in two separate cohorts. In the current study, which focuses on individual‐difference effects using a conventional general linear model (GLM) framework, the two cohorts were combined to maximize statistical power for examining associations between trait disgust and neural responses. The first experiment included 80 pictures that could elicit varying levels of subjective disgust experience from the DIRTI database (Haberkamp et al. 2017; 67 pictures), the IAPS database (Lang et al. 2008; 8 pictures), and the Nencki Affective Picture System (Marchewka et al. 2014; 5 pictures), distributed over four runs (with 20 stimuli in each run). To prevent category‐driven specificity in disgust, we selected a balanced sample from animals, scenes, and humans. Each picture was presented for 6 s; participants were instructed to look at the pictures and naturally experience the evoked emotion. After the presentation of each picture, a 2 s fixation cross was shown, followed by a 4 s response window during which participants rated their subjective disgust on a 5‐point Likert scale (1 = neutral; 5 = most intense disgust). Trials were separated by a jittered fixation cross epoch between 5 and 7 s. E‐Prime 2.0 was used to present the stimuli. The second experiment contained 60 pictures, all drawn from the DIRTI database (Haberkamp et al. 2017), distributed over two runs (with 30 stimuli per run). Each picture was displayed on the screen for 6 s, during which participants were required to pay attention to the pictures and naturally experience the elicited emotion. Then, a fixation cross jittered between 3 and 5 s was shown on the screen. Next, participants had 4 s to report the level of disgust they experienced for the stimuli on a 5‐point Likert scale (1 = neutral; 5 = most intense disgust). Trials were separated by a jittered fixation cross epoch between 5 and 7 s. E‐Prime 3.0 was used to present the stimuli. After data quality control (excluding participants with excessive head movement), the final sample comprised 142 participants, with 108 contributing data from the first experiment and 34 from the second experiment. Moreover, 133 out of 142 participants reported all five levels of subjective state disgust, whereas the remaining 9 reported ratings ranging only from level 1 to level 4. Before each experiment, participants performed a practice task outside the scanner to familiarize themselves with the experimental procedure. Both experiments reliably elicited the full range of subjective disgust ratings (Gan et al. 2024) (see Results for more information).

See Supporting Information Methods for the details of MRI data acquisition.

### 2.4 fMRI Data Preprocessing

SPM12 (Statistical Parametric Mapping, https://www.fil.ion.ucl.ac.uk/spm/software/spm12/) was used to preprocess and analyze the fMRI data. The initial five volumes of fMRI data from each run were discarded to allow for T1 equilibration. Before preprocessing, we identified global motion outliers within each run based on either of the following criteria: (1) signal intensity larger than three s.d. from the global mean or (2) signal intensity and Mahalanobis distances larger than 10 mean absolute deviations (https://github.com/canlab/CanlabCore). Each timepoint identified as outliers was subsequently incorporated into the first‐level model as a separate nuisance regressor.

The preprocessing pipeline encompassed several key steps, including slice timing correction to account for temporal discrepancies across slices, realignment to correct for head motion, and unwarping to compensate for distortions caused by magnetic field inhomogeneities. Subsequently, high‐resolution anatomical images underwent tissue segmentation into gray matter, white matter, cerebrospinal fluid, bone, fat, and air. The skull‐stripped and bias‐corrected structural image was then co‐registered to the functional data, followed by normalization of the functional images to the standard Montreal Neurological Institute (MNI) space, resampled to an isotropic voxel size of 2 × 2 × 2 mm^3^. Finally, spatial smoothing was applied using an 8‐mm full‐width at half‐maximum Gaussian kernel to enhance the signal‐to‐noise ratio and reduce spatial variability.

### 2.5 First‐Level fMRI Analysis

Following preprocessing, first‐level (subject‐specific) analysis was conducted using the GLM framework (Friston et al. 1994). Specifically, we constructed a GLM for each participant, which incorporated five or four regressors time‐logged to the presentations of pictures for each rating level (i.e., 1–5 for those whose ratings covered from 1 to 5, or 1–4 for those who did not report rating 5) and one boxcar regressor aligned with the timing of rating period, thus allowing for the separate modeling of neural activity in response to each disgust level and for the modeling of effects associated with motor processes, respectively. The fixation‐cross epoch served as an implicit baseline. Task‐related regressors were convolved with the canonical hemodynamic response function, and for each voxel a high‐pass filter with a cutoff of 128 s was implemented to remove low‐frequency signal drifts. The four (for the first experiment) or two (for the second experiment) fMRI task runs were concatenated for each participant using the SPM function spm_fmri_concatenate.m. Nuisance regressors comprised run‐specific intercepts, 24 motion parameters (six realignment parameters, their squares, derivatives, and squared derivatives per run), and vectors for outlier timepoints. Conditions of interest included high disgust (rating 4 and rating 5 for those whose subjective disgust ratings covered from 1 to 5, or rating 4 for those whose subjective disgust ratings only covered from 1 to 4), moderate disgust (rating 2 and rating 3), and neutral (rating 1). Contrast images between conditions (i.e., high disgust > neutral, high disgust > moderate disgust, and moderate disgust > neutral) used for the following regression analyses were estimated for each participant at each voxel, yielding statistical parametric maps that reflect the differential neural responses between conditions (Friston et al. 1994).

### 2.6 fMRI Regression Analysis

We employed a series of regression analyses to test whether variations in neural responses for each contrast were modulated by disgust propensity and disgust sensitivity. Because disgust propensity and disgust sensitivity represent conceptually related and substantially correlated (*r* = 0.71, *p* < 0.001; see Results) dimensions of trait disgust, entering both variables simultaneously into the same regression model would yield residualized regression coefficients that reflect only the variance unique to each construct after removal of shared variance (Wurm and Fisicaro 2014). Such coefficients may be difficult to interpret with respect to the original psychological constructs. Given that the primary aim of the present study was to characterize how each trait dimension—as typically conceptualized and measured in the literature—relates to disgust‐related neural processing, the two trait dimensions were therefore modeled separately in the regression analyses.

The resulting statistical maps were thresholded at *p* < 0.05 cluster‐level family‐wise error (FWE) correction, with cluster‐forming voxel‐level threshold at *p* < 0.001 (a minimum cluster size of 30 contiguous voxels was set to maintain spatial coherence), thereby obtaining regions significantly correlated with trait disgust scores.

In addition, to account for the rich trial‐wise variation in subjective ratings, we conducted a complementary parametric modulation analysis at the first level. The resulting parametric modulation maps were then entered into second‐level regression analyses with trait disgust measures (see Supporting Information Methods for details).

### 2.7 Mediation Analysis

To examine the relationship between state disgust, brain activation, and trait disgust, multiple mediation analyses were conducted via the SPSS Process v3.5. Of note, these analyses were exploratory in nature and mainly served to show a mediation within the disgust propensity association (details see Results). Here, state disgust was operationalized by the sum of participants' self‐reported subjective disgust ratings for each picture, brain activation was operationalized by the mean activation within each significant cluster derived from the above regression analyses (note that we only focused on those clusters that survived the stringent threshold, i.e., cluster‐level FWE *p* < 0.05 correction and voxel‐level *p* < 0.001; details see Results), trait disgust propensity was the sum of participants' ratings for the disgust propensity subscale of the Chinese version of DPSS‐R, and trait disgust sensitivity was the sum of participants' ratings for the disgust sensitivity subscale of the Chinese version of DPSS‐R. Mediation analysis aims to investigate whether the association between an independent variable (X) and a dependent variable (Y) can be explained by a third variable, known as the mediator (M). The X‐M relation, M‐Y relation, and X‐Y relation before and after controlling for the M are characterized by paths a, b, c, and c′, respectively. The total effect of X on Y (path c) is the sum of the direct effect (path c′) and the indirect (mediation) effect (the product of the path coefficients of paths a and b, i.e., a × b). Evidence for mediation is established when paths a, b, and a × b are all statistically significant. Moreover, when c′ is not significant, M plays a full mediation role; otherwise, M plays a partial mediation role. In this study, for each cluster we test whether disgust propensity (as well as disgust sensitivity) mediated the relationship between state disgust and the mean activation within this cluster. To evaluate the statistical significance of mediation effects, bootstrap tests with 10,000 iterations were employed. If the bootstrapped 99% confidence interval does not include zero, the effect will be considered significant (*p* < 0.001). Given that the two disgust‐induction fMRI tasks included differing numbers of pictures (i.e., 80 in the first task and 60 in the second task), directly combining the raw total scores of subjective disgust ratings across these tasks would be biased. Therefore, total scores were normalized within each task before aggregation to yield a standardized measure of state disgust.

### 2.8 Task‐Based Connectivity: MACM Analyses

To investigate the meta‐analytic co‐activation patterns of each significant region obtained from the above regression analyses (details see Results), MACM analyses were conducted using the peak coordinates of each cluster identified in the regression analyses with the stringent threshold (cluster‐level FWE *p* < 0.05 correction and voxel‐level *p* < 0.001; details see Results) as ROIs (with sphere radius = 6 mm) based on the BrainMap functional database. Briefly, MACM examines whole‐brain functional connectivity profiles by identifying the above‐chance covariance between two or more brain regions (Gan et al. 2022; Robinson et al. 2010). This approach provides a measure of task‐based functional connectivity by leveraging task‐related neuroimaging data (Langner et al. 2014).

We performed a series of MACM analyses utilizing Sleuth software (v.3.0.4) from the BrainMap platform (https://brainmap.org/sleuth/). The search terms in the Sleuth were specified as follows: (1) “Locations: MNI Image” to upload the respective spherical ROI in MNI space, (2) “Experiments: Activations, Activations only” to restrict results to activation data, (3) “Experiments: Context, Normal Mapping” to focus on normative tasks, (4) “Subjects: Diagnosis, Normals” to include only healthy participants. This enabled the detection of above‐chance co‐activation patterns within a given seed ROI across a wide range of neuroimaging experiments from the functional database. After each respective ROI search, eligible activation coordinates from experiments were converted into MNI space and exported as a text file. The MACM analyses were based on the two datasets for each of the ROIs. Specifically, the dataset with the right anterior insula as ROI included 42 experiments, 801 foci, and 718 participants; and the dataset with the left thalamus as ROI encompassed 17 experiments, 229 foci, and 288 participants. To determine areas of convergence of co‐activation with each ROI, these new datasets were analyzed separately using the activation likelihood estimation algorithm embedded in GingerALE v.3.0.2 (available via http://www.brainmap.org/ale/) with the following statistical criteria: cluster‐level FWE correction at *p* < 0.05, with an initial cluster‐forming threshold set at the voxel‐level of *p* < 0.001 (uncorrected) (Eickhoff et al. 2016; Ferraro et al. 2022; Gan et al. 2025, 2022; Klugah‐Brown et al. 2020, 2021), with *p* values generated by 5000 permutations. See Supporting Information Methods for the methodological overview of the activation likelihood estimation.

### 2.9 Task‐Free Connectivity: RSFC Analyses

To characterize intrinsic functional connectivity patterns of the regions identified through the above regression analyses (details see Results), RSFC analyses were performed on the original dataset, using 6 mm spherical ROIs centered on the peak coordinates of each significant cluster that survived the stringent threshold. Specifically, the original dataset used for the RSFC was from a separate cohort, consisting of 100 healthy subjects drawn from the Open Access Series of Imaging Studies (OASIS)‐3 dataset (https://central.xnat.org). See Supporting Information: Methods for the data acquisition parameters and preprocessing procedures of this dataset.

Using the Data Processing Assistant for Resting‐State fMRI (DPARSF, v.5.0_201001) (http://rfmri.org/DPARSF) toolbox, the preprocessed time series from each seed ROI was correlated with the time series of all other brain voxels. The resulting voxel‐wise correlation coefficients were transformed into Fisher's *z*‐scores and tested for consistency across participants (thresholded at *p* < 0.05 cluster‐level FWE correction, with cluster‐forming voxel‐level threshold at *p* < 0.001).

### 2.10 Consensus Connectivity Map

To identify overlapping connectivity patterns, MACM and RSFC results were integrated for each seed ROI using conjunction analyses based on the minimum statistic approach (Nichols et al. 2005). The resultant consensus connectivity maps identified brain regions showing consistent interactions with each seed across both task‐based and task‐independent functional connectivity profiles (Bore et al. 2024; Gan et al. 2025; Reimann et al. 2025).

### 2.11 Spatial Similarity Between Respective Maps and Thalamus Subregions

To pinpoint the contributions of different thalamus subregions to the regression map as well as to the consensus connectivity maps (details see Results), we employed a sensitive approach that illustrates spatial similarity between respective maps and eight thalamus subregions derived from the Human Brainnetome Atlas (Fan et al. 2016). Consistent with recent studies (Čeko et al. 2022; Gan et al. 2024; Zhang, Qing, et al. 2025), spatial similarity was computed as cosine similarity between each thalamus subregion and the corresponding map obtained from regression and connectivity analyses.

## 3 Results

### 3.1 Self‐Report Data

**FIGURE 1:**
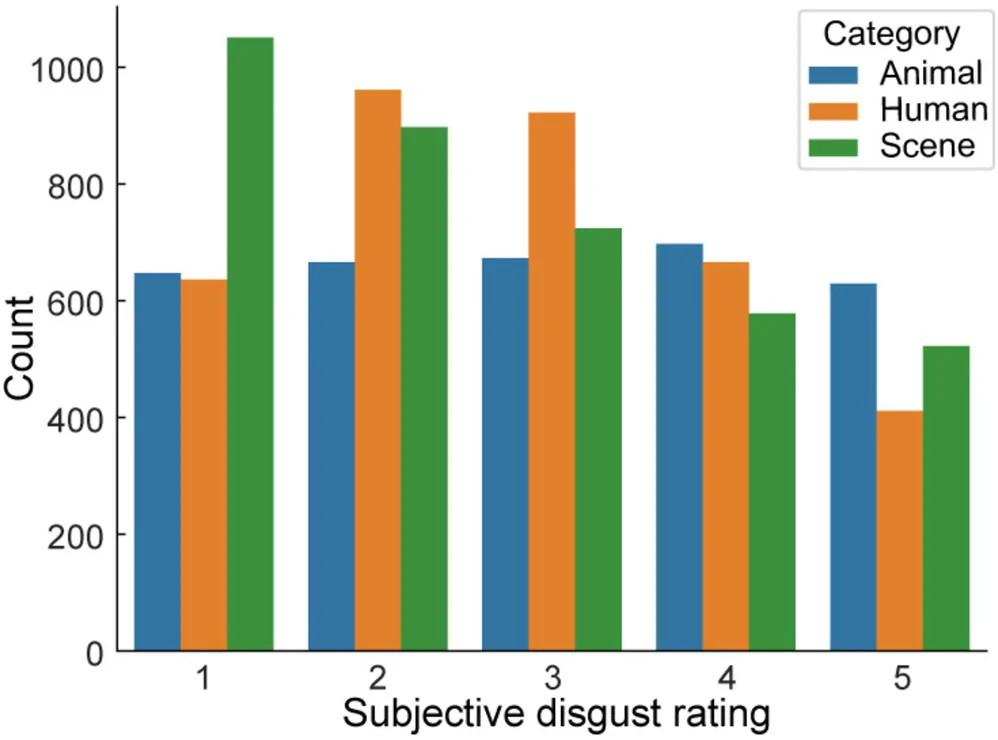
The distribution of state disgust ratings for each disgust category (animal, human, and scene).

During fMRI, state disgust was evoked using validated disgust‐specific affective pictures (the DIRTI database, Haberkamp et al. 2017). These pictures successfully elicited sufficient levels of subjective state disgust across the most common disgust‐related categories (animal, human, and scene; Figure 1), with over 11% of stimuli in each category inducing the highest level of state disgust (rating 5).

Disgust propensity and disgust sensitivity were highly correlated (*r* = 0.71, *p* < 0.001), consistent with the original English version of DPSS (*r* = 0.59, *p* < 0.01; details see van Overveld et al. 2006). Moreover, both disgust propensity (*r* = 0.28, *p* < 0.001) and disgust sensitivity (*r* = 0.33, *p* < 0.001) were positively correlated with the experienced state disgust elicited by disgust stimuli.

Considering that prior research showed sex differences in disgust propensity (Caseras et al. 2007), an independent samples *t*‐test was performed to compare disgust propensity between females and males. However, there were no significant sex differences in disgust propensity (female: 24.20 ± 4.43; male: 23.73 ± 5.08; *t*_(140)_ = 0.59, *p* = 0.56). Furthermore, we did not find sex differences in either disgust sensitivity (female: 18.60 ± 5.08; male: 19.05 ± 5.35; *t*_(140)_ = 0.51, *p* = 0.61) or the total scale score (female: 42.80 ± 8.65; male: 42.78 ± 9.82; *t*_(140)_ = 0.02, *p* = 0.99). In addition, females and males did not differ in state disgust (female: −0.11 ± 0.96 after normalization; male: 0.13 ± 1.02 after normalization; *t*_(140)_ = 1.46, *p* = 0.15).

To examine whether individual differences in reaction time might be related to disgust propensity as well as disgust sensitivity, we calculated correlations between reaction time measures for different rating levels and both disgust propensity and disgust sensitivity. None of these correlations reached significance (see Supporting Information Results for details).

### 3.2 Regression Results

All regression analyses were initially assessed using a cluster‐level FWE correction (*p* < 0.05) with a cluster‐forming threshold of voxel‐level *p* < 0.001. Under this threshold, significant effects were observed only for the high disgust > neutral contrast. For the contrasts high disgust > moderate disgust and moderate disgust > neutral, no clusters survived FWE correction. Therefore, results for these contrasts are additionally reported at a voxel‐level threshold of *p* < 0.001 (uncorrected) for exploratory purposes and should be interpreted with caution.

**FIGURE 2:**
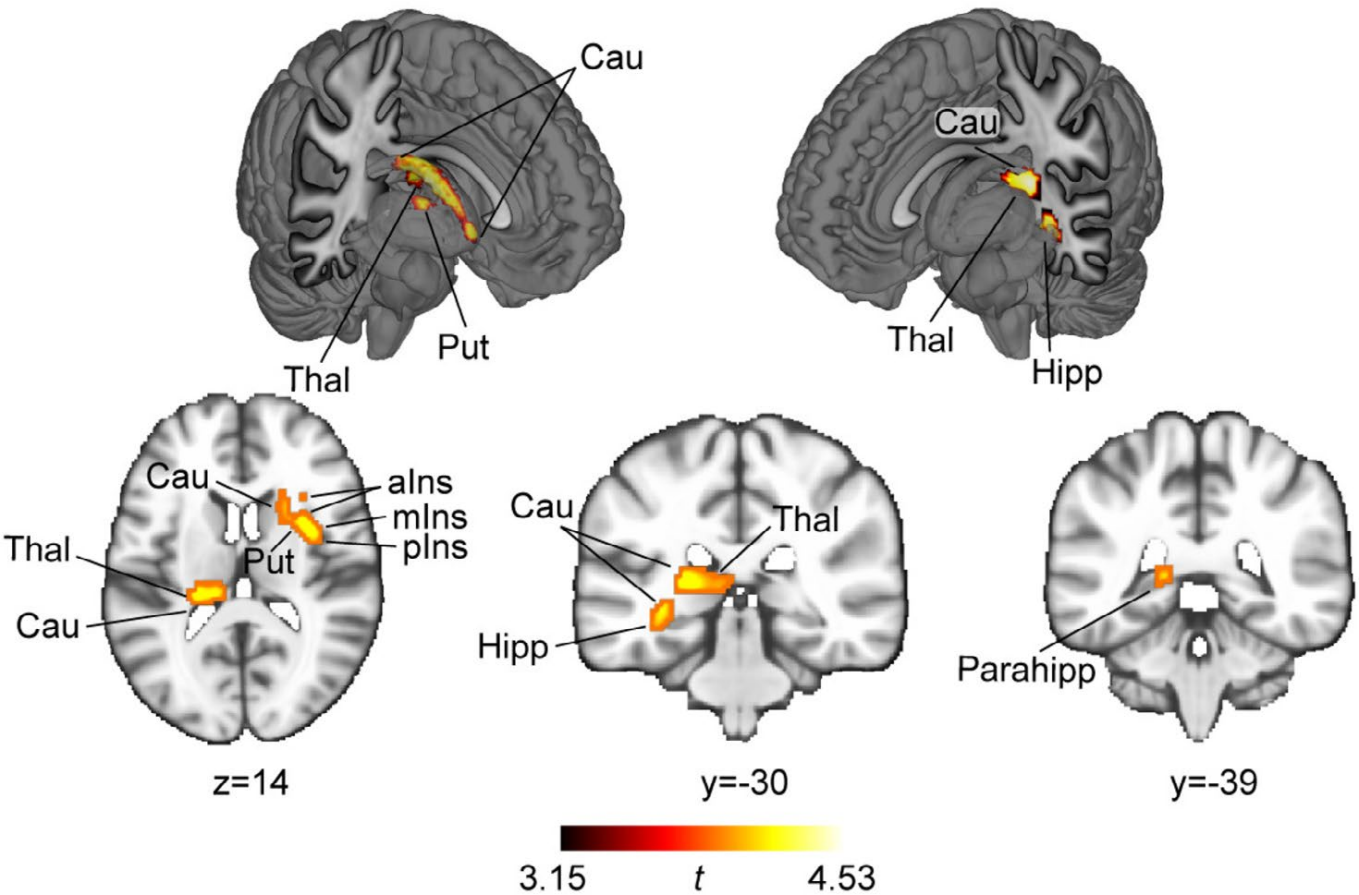
Positive association between disgust propensity scores and brain activation while viewing high disgust stimuli (high disgust > neutral). Activated voxel group shown at a statistical threshold of voxel‐level *p* < 0.001 and cluster‐level FWE *p* < 0.05 correction. Abbreviations: AIns, anterior insula; Cau, caudate; Hipp, hippocampus; mIns, middle insula; Parahipp, parahippocampal gyrus; pIns, posterior insula; Put, putamen; Thal, thalamus.

Specifically, whole‐brain regression analysis showed that activation to high disgust stimuli (high disgust > neutral) was significantly modulated by participants' disgust propensity scores in a cluster located in the right anterior insula, encompassing regions of the anterior, mid‐ and posterior insular cortex, caudate, and putamen as well as a cluster located in the left thalamus, encompassing regions of the thalamus, hippocampus, caudate, and parahippocampal gyrus (Table 1 and Figure 2). In each of these regions, higher trait disgust propensity was associated with increased neural activation in response to high disgust stimuli. We did not find significant negative associations between participants' disgust propensity scores and brain activation. Results remained stable when including sex and the reaction time difference for high disgust versus neutral pictures as covariates in the regression model (details see Table S1 and Figure S1).

**TABLE 1:** Brain areas where activation to viewing high disgust stimuli (high disgust > neutral) was significantly associated with participants' scores on the disgust propensity subscale.

Regions	Side	MNI peak coordinates (x, y, z)			*t*‐value	Cluster size	Cluster‐FWE
Anterior insula, middle insula, posterior insula, caudate, putamen	R	32	4	14	4.43	494	0.013
Thalamus, hippocampus, caudate, parahippocampal gyrus	L	−24	−32	10	4.53	363	0.042

*Note:* voxel‐level uncorrected *p* < 0.001, combined with cluster‐level FWE correction.

Abbreviations: L, left; R, right.

As for the condition of high disgust > moderate disgust, consistent with the findings reported above, activation in the right anterior insula and left thalamus clusters was again observed. Increased activation was also found in bilateral occipital cortices and left inferior/middle frontal gyrus (Supporting Information: Results, Table S2, and Figure S2). For the condition of moderate disgust > neutral, significant modulation by participants' disgust propensity scores was observed in a set of frontal and motor regions (Supporting Information: Results, Table S3, and Figure S3). It should be noted that these results are suggestive due to the lenient threshold. Results remained stable when incorporating sex and the reaction time difference between high disgust and moderate disgust pictures, as well as between moderate disgust and neutral pictures, as covariates.

However, regression analyses across the high disgust > neutral, high disgust > moderate disgust, and moderate disgust > neutral contrasts failed to identify any brain regions whose activation was significantly modulated by participants' disgust sensitivity scores, either under the stringent or liberal threshold. These findings remained unchanged when sex and the reaction time difference between contrasts were included as covariates.

Complementary analyses based on parametric modulation of trial‐wise subjective ratings revealed that disgust propensity was associated with neural responses in regions including the anterior, mid‐ and posterior insula, thalamus, and striatal and medial temporal areas; however, these effects were observed only at a voxel‐level threshold (*p* < 0.001, uncorrected) and were therefore reported as exploratory (see Table S4 and Figure S4). No significant associations were observed with disgust sensitivity, even at this liberal threshold.

### 3.3 Mediation Results

Regression analysis identified two clusters (right anterior insula and left thalamus; high disgust > neutral, cluster‐level FWE *p* < 0.05, voxel‐level *p* < 0.001, Table 1) with activation significantly associated with disgust propensity. Next, we performed a series of mediation analyses to test whether trait disgust mediated the relationship between state disgust and the mean activation in each cluster.

**FIGURE 3:**
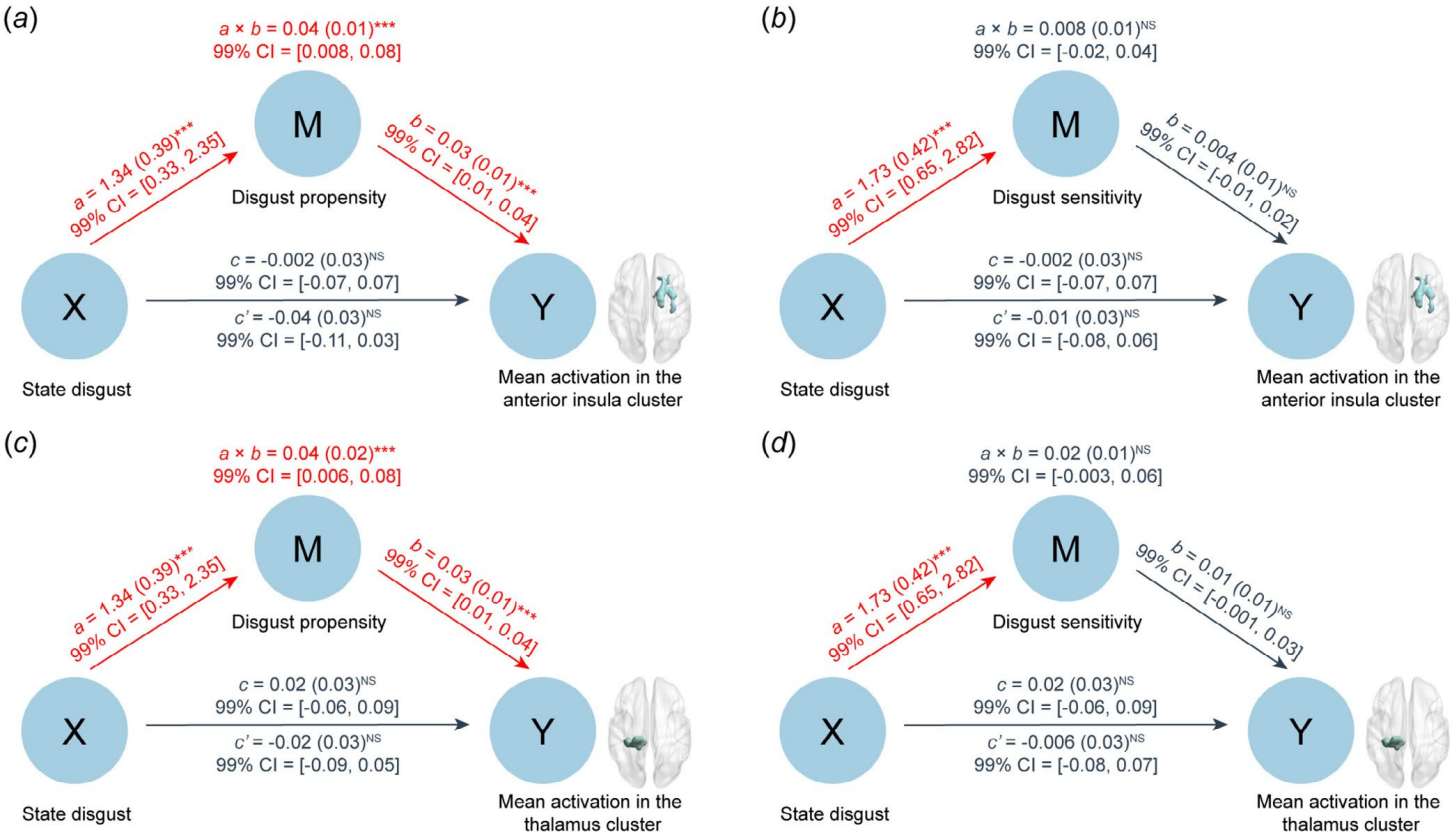
Mediation analyses exploring whether trait disgust mediates the relationship between state disgust and the mean activation in the anterior insula and thalamus clusters. (a) Disgust propensity fully mediates the association between state disgust and the mean activation of the anterior insula cluster. (b) Disgust sensitivity does not mediate the effect of state disgust on the mean activation of the anterior insula cluster. (c) Disgust propensity fully mediates the association between state disgust and the mean activation of the thalamus cluster. (d) Disgust sensitivity fails to mediate the effect of state disgust on the mean activation of the thalamus cluster. ***p < 0.001, NS not significant.

We first tested whether disgust propensity (*M*) could mediate the relationship between state disgust (*X*) and the mean activation of the anterior insula cluster (*Y*). As shown in Figure 3a, state disgust was positively correlated with disgust propensity (path a; *β* = 1.34, SE = 0.39, 99% CI = [0.33, 2.35], *p* < 0.001), disgust propensity was positively associated with the mean activation of the anterior insula cluster independently of state disgust (path b; *β* = 0.03, SE = 0.01, 99% CI = [0.01, 0.04], *p* < 0.001), and there was a significant mediation effect (path a × b; *β* = 0.04, SE = 0.01, 99% CI = [0.008, 0.08], *p* < 0.001). Furthermore, the total effect (path c; *β* = −0.002, SE = 0.03, 99% CI = [−0.07, 0.07], *p* = 0.93) and the non‐mediated effect (path c′; *β* = −0.04, SE = 0.03, 99% CI = [−0.11, 0.03], *p* = 0.13) were not significant. These results indicate that disgust propensity plays a full mediation role in the effect of state disgust on the mean activation of the anterior insula cluster. We next replaced disgust propensity with disgust sensitivity and ran a second mediation analysis. However, the results revealed that disgust sensitivity could not mediate the association between state disgust and the mean activation of the anterior insula cluster (Figure 3b).

We continued to examine whether disgust propensity (*M*) could explain the relationship between state disgust (*X*) and the mean activation of the thalamus cluster (*Y*). According to Figure 3c, the results showed that state disgust had a positive association with disgust propensity (path a; *β* = 1.34, SE = 0.39, 99% CI = [0.33, 2.35], *p* < 0.001), disgust propensity was positively correlated with the mean activation of the thalamus cluster (path b; *β* = 0.03, SE = 0.01, 99% CI = [0.01, 0.04], *p* < 0.001), and there was a significant mediation effect (path a × b; *β* = 0.04, SE = 0.02, 99% CI = [0.006, 0.08], *p* < 0.001). Moreover, the total effect (path c; *β* = 0.02, SE = 0.03, 99% CI = [−0.06, 0.09], *p* = 0.53) and the non‐mediated effect (path c′; *β* = −0.02, SE = 0.03, 99% CI = [−0.09, 0.05], *p* = 0.48) were not significant. These results indicate that disgust propensity plays a full mediation role in the effect of state disgust on the mean activation of the thalamus cluster. We next constructed another mediation model with disgust sensitivity as *M*, state disgust as *X*, and the mean activation of the thalamus cluster as *Y*. Nevertheless, the results showed that disgust sensitivity failed to mediate the effect of state disgust on the mean activation of the thalamus cluster (Figure 3d).

Notably, incorporating the mean activation in each cluster from the regression results after including sex and the reaction time difference for high disgust versus neutral pictures as covariates in the mediation model (i.e., Table S1) did not affect the pattern of results (Figure S5).

### 3.4 Consensus Connectivity Maps of MACM and RSFC Profiles

**FIGURE 4:**
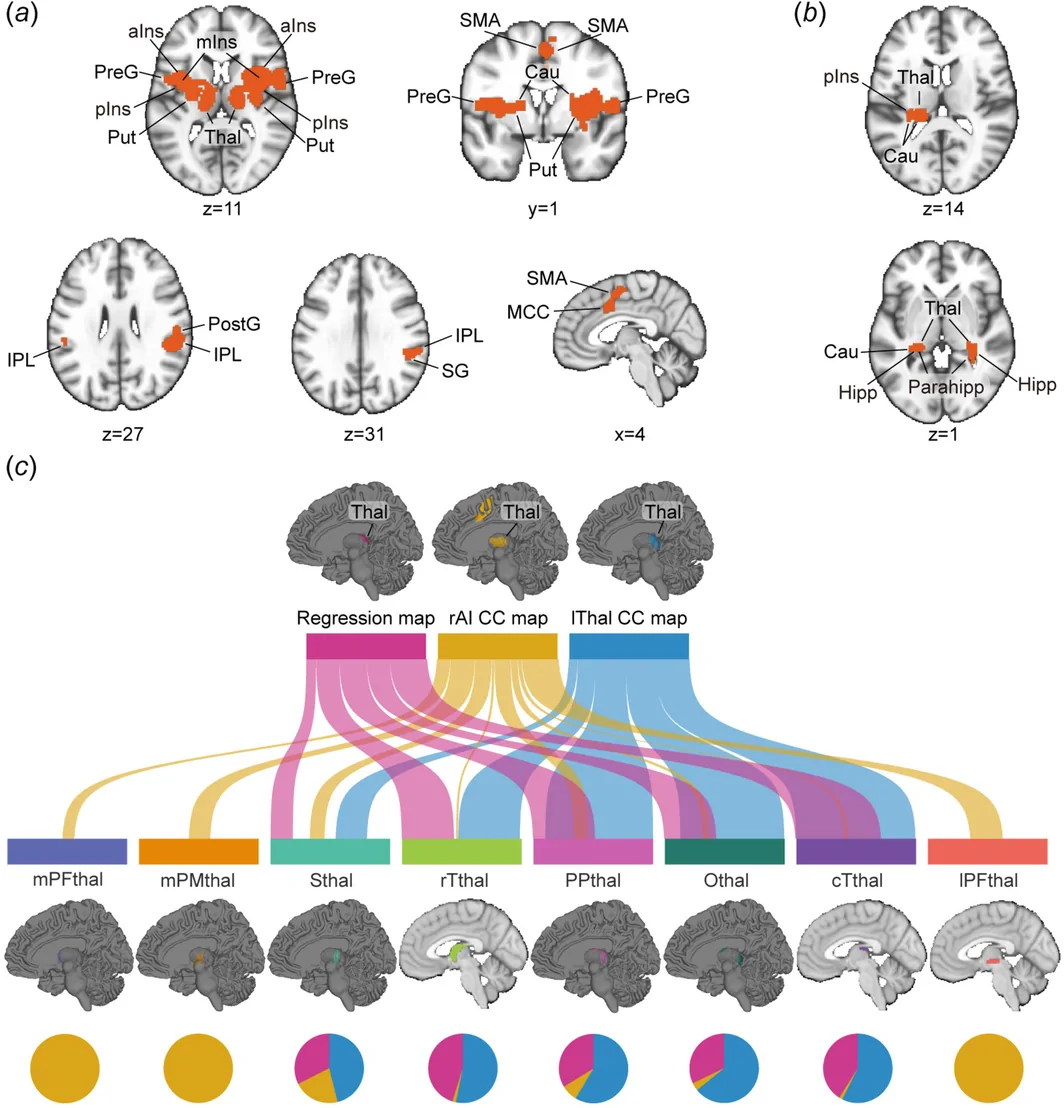
Consensus connectivity networks for the anterior insula and thalamus from the regression analysis. (a) Results of the consensus connectivity map for the right anterior insula. (b) Results of the consensus connectivity map for the left thalamus. (c) River plots showing spatial similarity (cosine similarity) between respective maps (i.e., regression map, Figure 2; the consensus connectivity map of the right anterior insula, Figure 4a; the consensus connectivity map of the left thalamus, Figure 4b) and eight thalamus subregions. Ribbons are normalized by the max cosine similarity across all eight thalamus subregions. Ribbon locations in relation to the boxes are arbitrary. Pie charts show the relative contributions of each map to the thalamus subregion (i.e., percentage of voxels with the highest cosine similarity for each map). Abbreviations: AIns, anterior insula; Cau, caudate; cTthal, caudal temporal thalamus; Hipp, hippocampus; IPL, inferior parietal lobule; lPFthal, lateral prefrontal thalamus; lThal CC map, left thalamus consensus connectivity map; MCC, midcingulate cortex; mIns, middle insula; mPFthal, medial prefrontal thalamus; mPMthal, pre‐motor thalamus; Othal, occipital thalamus; Parahipp, parahippocampal gyrus; pIns, posterior insula; PostG, Postcentral Gyrus; PPthal, posterior parietal thalamus; PreG, Precentral Gyrus; Put, putamen; rAI CC map, right anterior insula consensus connectivity map; rTthal, rostral temporal thalamus; SG, supramarginal gyrus; SMA, supplementary motor area; Sthal, sensory thalamus; Thal, thalamus.

The consensus connectivity map of the right anterior insula from the regression analysis was determined based on the connectivity profiles of the MACM and RSFC analyses. This analysis yielded a consensus connectivity network of the right anterior insula during both task and resting states, including bilateral anterior and mid‐ and posterior insular cortex, putamen, caudate, thalamus, and right midcingulate cortex. Moreover, it also contained motor‐related regions such as the supplementary motor area, primary motor cortex (e.g., precentral gyrus), and parietal motor regions (e.g., inferior parietal lobule, postcentral gyrus, and supramarginal gyrus) (Figure 4a, Figure S6, and Table S5).

Identifying the consensus connectivity map of the left thalamus from the regression analysis showed convergence at the bilateral thalamus, hippocampus, parahippocampal gyrus, left caudate, and left posterior insula (Figure 4b, Figure S7, and Table S6).

The following spatial similarity analyses between eight thalamus subregions and the regression map (i.e., Figure 2) as well as the consensus connectivity maps (i.e., Figure 4a,b) revealed that the medial prefrontal thalamus, pre‐motor thalamus, and lateral prefrontal thalamus uniquely contributed to the consensus connectivity map of the right anterior insula; the sensory thalamus, rostral temporal thalamus, posterior parietal thalamus, occipital thalamus, and caudal temporal thalamus contained significant voxels across all three maps, whereas the latter four thalamus subregions were more strongly involved in the regression map and the consensus connectivity map of the left thalamus (Figure 4c).

## 4 Discussion

The current study examined whether individual differences in disgust propensity and disgust sensitivity modulate neural responses to disgust‐evoking stimuli, using statistically rigorous whole‐brain regression analyses in a large, well‐powered sample. To account for variations in state disgust, we constructed multiple contrast images reflecting different levels of evoked disgust (e.g., high > neutral, moderate > neutral), which were then entered into separate regression models alongside trait‐level measures, thereby allowing us to better isolate their respective modulatory effects on disgust‐related neural circuitry. The regression analyses revealed a differential pattern of associations between the two trait dimensions, with higher levels of disgust propensity—but not variations in disgust sensitivity—being associated with higher disgust‐related brain activity in the right insular cortex and adjacent dorsal striatum as well as the left thalamus, caudate, and adjacent (para‐)hippocampal formation. Further support for the critical role of these brain systems and the differentiation between the disgust traits was provided by mediation analyses showing that disgust propensity, but not disgust sensitivity, played a full mediation role in the effect of subjective disgust experience (state disgust) on the activation of the anterior insula and thalamic clusters, both of which were identified in the whole‐brain regression analyses. Additional network‐level results indicated that the two identified regions in the insular cortex and thalamus communicate with distinguishable yet interacting brain systems, with the insular systems engaging with the dorsal striatum, midcingulate cortex, motor‐related frontal and parietal regions as well as the thalamus and the thalamic cluster interacting with the posterior insula, (para‐)hippocampal formation as well as the caudate. Together, these findings underscore that individual differences in disgust propensity, but not disgust sensitivity, modulate neurofunctional reactivity toward disgust stimuli and mediate the association between neural reactivity in these regions and subjective disgust experience.

The regression analysis revealed that activation in the right anterior insula cluster (including the anterior, mid‐, and posterior insula, caudate, and putamen) and the left thalamus cluster (encompassing the thalamus, hippocampus, parahippocampal gyrus, and caudate) showed positive correlations with disgust propensity scores while viewing high disgust stimuli (high disgust > neutral). These findings are in line with our a priori hypotheses regarding the involvement of insular and subcortical regions. Among these regions, the insula has been repeatedly defined as a core region in disgust processing. For example, evidence from lesion models (Adolphs et al. 2003; Calder et al. 2000; Cantone et al. 2019; Holtmann et al. 2020; Kipps et al. 2007), intracerebral recording studies (e.g., see Krolak‐Salmon et al. 2003), and neuroimaging studies in healthy populations with multimodal disgust induction paradigms (e.g., visual, gustatory, olfactory, and imagination Corradi‐DellAcqua et al. 2016; Gan et al. 2025; Gan et al. 2022; Heining et al. 2004; Jabbi et al. 2008; Wicker et al. 2003) converges to support its role in disgust processing, even though it is a functionally heterogeneous region (Ferraro et al. 2022; Li et al. 2019; Molnar‐Szakacs and Uddin 2022; Uddin 2015; Zhou et al. 2020). Similarly, basal ganglia regions such as the dorsal striatum, including the caudate and putamen, have been extensively associated with the neural processing of disgust, both in lesion (Calder et al. 2000; Gray et al. 1997; Paulmann et al. 2009) and multimodal (recall, visual, and gustatory, etc. Fitzgerald et al. 2004; Jabbi et al. 2008; Mataix‐Cols et al. 2008) contexts. Although less frequently reported as key disgust‐related systems compared to the insula and basal ganglia, recent meta‐analyses have confirmed the role of the parahippocampal gyrus in the experience of disgust (Gan et al. 2025, 2022). The thalamus has not received attention in previous studies on disgust, yet recent studies demonstrate an involvement of the thalamus in anxious arousal (Liu et al. 2024) and in mediating both subjective experience and autonomous physiological reactivity during affective arousal (Zhang, Gan, et al. 2025). Finally, the hippocampus showed a higher reactivity as a function of disgust propensity and, together with the parahippocampal gyrus, may reflect a link between the current emotional experience and memory processing, including valence‐ and arousal‐facilitated mnemonic encoding or an integration of the current affective experience with previous experiences (e.g., see Pacheco‐Estefan et al. 2025; Qasim et al. 2023; Xu et al. 2022). Notably, all these regions observed in the regression analyses have been demonstrated to be associated with and predictive of subjective disgust experience (Gan et al. 2024). Overall, these findings may reflect that individual differences in trait disgust propensity modulate neurofunctional reactivity to disgust‐inducing stimuli in systems related to the affective experience of disgust and arousal, their interactions with memory, as well as the hard‐wired autonomous and motor‐preparation responses supporting defensive avoidance reactions.

Further network‐level analyses were utilized to determine the networks that the anterior insula and thalamus systems communicate with using a combination of task‐based and intrinsic connectivity indices. The consensus connectivity map of the right anterior insula was coupled not only with regions observed in the main regression results (i.e., anterior, mid‐, and posterior insular cortex, as well as putamen and caudate), but also with regions such as the thalamus, midcingulate cortex, and a set of motor‐related regions (e.g., the supplementary motor area, primary motor cortex, inferior parietal lobule, postcentral gyrus, and supramarginal gyrus). The spatial similarity analyses enabled us to dissect the thalamus in a more nuanced way, revealing that the thalamus in the consensus connectivity map of the right anterior insula primarily comprised the following subparts: medial prefrontal thalamus, lateral prefrontal thalamus, pre‐motor thalamus, and sensory thalamus. Functional interactions between these regions may suggest the existence of a defensive‐avoidance neural circuit that is modulated by disgust propensity. The insula and midcingulate cortex present core nodes of the salience network and may reflect the high salience of disgust‐related processes (Gan et al. 2025), potentially engaging the cortical motor‐related regions and subcortical pre‐motor thalamus, and, via the basal ganglia and thalamus pathway, initiating goal‐directed defensive‐avoidance behavior (e.g., withdrawal and inhibition) (Holtmann et al. 2020; Liu, Lai, et al. 2025; Mair et al. 2022; Martinez‐Garcia et al. 2020). The consensus connectivity map of the left thalamus mirrored the regions identified in the core regression analysis (i.e., thalamus, hippocampus, parahippocampal gyrus, and caudate), and additionally encompassed the posterior insula. Interestingly, spatial similarity analysis revealed that the thalamic subregions in the consensus connectivity map of the left thalamus differed substantially from those observed in the consensus connectivity map of the right anterior insula, but closely matched those identified in the regression analysis (e.g., sensory thalamus, posterior parietal thalamus, occipital thalamus, and rostral and caudal temporal thalamus—with the latter four being largely unique to the regression map and the consensus connectivity map of the left thalamus), suggesting strong convergence between trait‐linked activation and its connectivity architecture. The above regions observed in the consensus connectivity map of the left thalamus may indicate that the two identified systems centered around the insula and thalamus serve distinct subfunctions in disgust processing, but also converge on the sensory thalamus and posterior insula as relay stations vital for sensory processing (Alonso‐Matielo et al. 2023; Torrico and Munakomi 2023). Thalamic activity varies with arousal intensities (Anders et al. 2004) and may initiate autonomic and defensive responses toward potential threats (Gan et al. 2024; Zhang, Gan, et al. 2025); the hippocampus and parahippocampal gyrus may support the integration of the momentary experience with personal and contextual memories related to disgust (Pacheco‐Estefan et al. 2025; Qasim et al. 2023; Tao et al. 2021; Xu et al. 2022); and the engagement of the caudate and posterior insula likely reflects their roles in constructing subjective disgust experience.

The regression analysis incorporating disgust propensity as a covariate for the contrast of high disgust > moderate disgust revealed a set of regions largely overlapping with those observed in the contrast of high disgust > neutral (e.g., the right anterior and left thalamus clusters), while moreover extending to visual and frontal areas. The regression analysis for the moderate disgust > neutral contrast showed that disgust propensity significantly modulated activity in frontal and motor‐related regions. Of note, these results are suggestive and should be interpreted cautiously, given the relatively lenient threshold applied (i.e., voxel‐level uncorrected *p* < 0.001). These findings also underline the importance of incorporating individual differences in subjective (state) disgust responses, as standard categorizations of stimuli (e.g., “high disgust”) may not reflect how strongly each participant actually experiences them (Čeko et al. 2022; Chang et al. 2015; Stark et al. 2007; Zhang, Gan, et al. 2025; Zhou et al. 2021). Ignoring such variability may blur the mapping between subjective affect and brain activity, and limit our understanding of how trait disgust shapes the emotional neural circuit.

In contrast, no significant effects between disgust sensitivity and disgust‐associated brain activity were observed, although on the behavioral level both disgust propensity (*r* = 0.28, *p* < 0.001) and disgust sensitivity (*r* = 0.33, *p* < 0.001) were positively correlated with subjective disgust experience, indicating that behavioral data alone cannot fully distinguish their contributions. This pattern suggests that disgust propensity and disgust sensitivity may differentially relate to neural processes underlying disgust. Disgust propensity is generally conceptualized as the tendency to experience disgust more readily, and may therefore be more directly linked to stimulus‐driven neural responses in disgust‐related regions. In contrast, disgust sensitivity reflects the extent to which individuals perceive the experience of disgust as unpleasant, and may be more closely related to higher‐order evaluative or regulatory processes that are not fully captured by the present task design. It is also possible that disgust sensitivity operates over different temporal dynamics, potentially influencing later stages of emotional processing rather than the early stimulus‐evoked responses examined here. In addition, disgust sensitivity may partially overlap with broader affective traits such as general anxiety, which could reduce its specificity in predicting neural activity within disgust‐related circuits.

Notably, exploratory parametric modulation analyses yielded a largely overlapping pattern of regions (e.g., insula, thalamus, striatum, and (para‐)hippocampal formation) for disgust propensity, although these effects did not survive cluster‐level correction. In contrast, no significant associations were observed for disgust sensitivity, even at a liberal threshold. This pattern provides tentative support for the robustness of the observed propensity‐related effects, while also highlighting the need for cautious interpretation.

The exploratory mediation analyses showed that it was disgust propensity, rather than disgust sensitivity, that fully mediated the relationship between state disgust and brain responses during disgust‐related affective processing. These results underscore a close association between trait‐level disgust and both the neural and behavioral variations, indicating that the brain responses associated with propensity were in turn related to the actual subjective feeling of disgust. Complementary evidence for the above findings comes from a previous study capitalizing on reverse inference, which demonstrated that the neural signature of subjective disgust experience mediated the influence of the general negative signature on state disgust ratings, but not vice versa (Gan et al. 2024). Notably, this study was conducted using the same data as the present work.

The differential pattern of neural associations between disgust sensitivity and disgust propensity resonates with previous results indicating that the two disgust traits differ in their associations with experience of actual disgust, general negative emotional sensitivity, disgust‐related avoidance, and anxiety disorder symptoms (Cisler et al. 2009; Davey 2011; Goetz et al. 2013; Olatunji et al. 2010, 2011, 2009; van Overveld et al. 2010, 2006).

The findings of this study may also contribute to a better understanding of psychiatric disorders characterized by altered disgust reactivity and disgust‐related learning, such as obsessive‐compulsive disorder (OCD) (Rast et al. 2023; Wang et al. 2024). On the behavioral level, trait disgust propensity may serve as a psychological marker for monitoring or predicting treatment response in OCD. For instance, Olatunji et al. demonstrated that the reduction in disgust propensity over time mediated improvement in OCD symptoms, even when improvement in negative affect was taken into account (Olatunji et al. 2011). On the neural level, some of the brain regions identified in the present study may represent candidate neural targets for interventions aimed at modulating pathological disgust responses. For example, enhanced activation of the insula cortex and dorsal striatum during processing and generalization of disgust stimuli has been reported in OCD (Liu, Wang, et al. 2025; Shapira et al. 2003; Vicario et al. 2017).

Although the present study employed whole‐brain regression analyses with stringent threshold corrections and further incorporated mediation analyses to provide tentative mechanistic insights, these methods are inherently correlational in nature and do not permit definitive causal inference. To more robustly establish causal links between trait disgust and neural responses, future research may benefit from noninvasive brain stimulation techniques (e.g., transcranial magnetic stimulation), longitudinal tracking of trait and neural dynamics, or randomized intervention designs targeting trait‐level disgust modulation. Moreover, the present sample consisted exclusively of Chinese young adults. Cultural factors may influence the elicitation and regulation of disgust responses, and thus the extent to which the present findings generalize to other cultural contexts or age groups remains to be established. Future studies including more diverse samples will be important for determining whether the observed associations between disgust propensity and neural responses to disgust‐inducing stimuli generalize beyond East Asian university populations.

## 5 Conclusion

Previous studies have shown inconsistent results with respect to neural variations related to trait disgust propensity and disgust sensitivity. As the first study to examine the modulatory role of trait disgust on neural responses across varying levels of induced disgust, the current work demonstrates that disgust propensity, rather than disgust sensitivity, robustly modulates neural responses to disgust‐inducing stimuli in regions consistently implicated in this emotion. These findings also shed light on the newly established association between heightened disgust propensity and intensified experience of state disgust, by identifying the neural circuit underlying this effect—encompassing the insula, dorsal striatum, thalamus, parahippocampal gyrus, and hippocampus—a network of regions previously linked to defensive and avoidance‐related responses to highly aversive stimuli. Furthermore, the mediation and network‐level results offer converging support for the above pattern, providing additional insight into how trait disgust shapes affective neural mechanisms. Ultimately, these findings may inform the development of targeted interventions for disgust‐related mental disorders.

## Funding

This work was supported by the Ministry of Science and Technology of China (STI 2030–Major Projects 2022ZD0208500), National Natural Science Foundation of China (NSFC 82271583), the Hong Kong University Grants Council (GRF 17615525), the University of Hong Kong seed funding and start‐up schemes (2407102536).

## Conflicts of Interest

The authors declare no conflicts of interest.

## Supporting information


**Table S1:** Brain areas where activation to viewing high disgust stimuli (high disgust > neutral) was significantly associated with participants' scores on the disgust propensity subscale (further including sex and the reaction time difference for high disgust versus neutral pictures as covariates in the regression analysis).
**Table S2:** Brain areas where activation to viewing high disgust stimuli (high disgust > moderate disgust) was significantly associated with participants' scores on the disgust propensity subscale.
**Table S3:** Brain areas where activation to viewing moderate disgust stimuli (moderate disgust > neutral) was significantly associated with participants' scores on the disgust propensity subscale.
**Table S4:** Brain regions where parametric modulation of neural responses by subjective disgust ratings was significantly associated with participants' scores on the disgust propensity subscale.
**Table S5:** Conjunction analysis of MACM and RSFC results for the right anterior insula from regression analysis.
**Table S6:** Conjunction analysis of MACM and RSFC results for the left thalamus from regression analysis.
**Figure S1:** Positive association between disgust propensity scores and brain activation while viewing high disgust stimuli (high disgust > neutral), with sex and the reaction time difference for high disgust versus neutral pictures as covariates. Note: activated voxel group shown at a statistical threshold of voxel‐level *p* < 0.001 and cluster‐level *p* < 0.05. Abbreviations: aIns, anterior insula; Cau, caudate; Hipp, hippocampus; mIns, middle insula; Parahipp, parahippocampal gyrus; pIns, posterior insula; Put, putamen; Thal, thalamus.
**Figure S2:** Positive association between disgust propensity scores and brain activation while viewing high disgust stimuli (high disgust > moderate disgust). Note: activated voxel group shown at a statistical threshold of voxel‐level *p* < 0.001. Abbreviations: aIns, anterior insula; Cau, caudate; Hipp, hippocampus; IFG, inferior frontal gyrus; IOG, inferior occipital gyrus; LG, lingual gyrus; MFG, middle frontal gyrus; mIns, middle insula; MOG, middle occipital gyrus; Parahipp, parahippocampal gyrus; pIns, posterior insula; Put, putamen; Thal, thalamus.
**Figure S3:** Positive association between disgust propensity scores and brain activation while viewing moderate disgust stimuli (moderate disgust > neutral). Note: activated voxel group shown at a statistical threshold of voxel‐level *p* < 0.001. Abbreviations: MFG, middle frontal gyrus; PL, paracentral lobule; PostG, postcentral gyrus; PreG, precentral gyrus; SPG, superior frontal gyrus.
**Figure S4:** Positive association between disgust propensity and parametric modulation of neural responses by subjective disgust ratings. Note: activated voxel group shown at a statistical threshold of voxel‐level *p* < 0.001. Abbreviations: aIns, anterior insula; Cau, caudate; Hipp, hippocampus; IPL, inferior parietal lobule; mIns, middle insula; Parahipp, parahippocampal gyrus; pIns, posterior insula; PostG, postcentral gyrus; STG, superior temporal gyrus; Thal, thalamus.
**Figure S5:** Mediation analyses exploring whether trait disgust mediates the relationship between state disgust and the mean activation (from regression results that included sex and the reaction time difference for high disgust versus neutral pictures as covariates) in the anterior insula and thalamus clusters. (a) Disgust propensity fully mediates the association between state disgust and the mean activation of the anterior insula cluster. (b) Disgust sensitivity does not mediate the effect of state disgust on the mean activation of the anterior insula cluster. (c) Disgust propensity fully mediates the association between state disgust and the mean activation of the thalamus cluster. (d) Disgust sensitivity fails to mediate the effect of state disgust on the mean activation of the thalamus cluster. ***p < 0.001, NS not significant.
**Figure S6:** Connectivity profiles of the right anterior insula from regression analysis. (a) Task‐based connectivity map (i.e., MACM). (b) Task‐free connectivity map (i.e., RSFC). (c) Consensus connectivity map (i.e., MACM ∩ RSFC, where ∩ indicates conjunction). Abbreviation: ALE, activation likelihood estimation.
**Figure S7:** Connectivity profiles of the left thalamus from regression analysis. (a) Task‐based connectivity map (i.e., MACM). (b) Task‐free connectivity map (i.e., RSFC). (c) Consensus connectivity map (i.e., MACM ∩ RSFC, where ∩ indicates conjunction). Abbreviation: ALE, activation likelihood estimation.

## Data Availability

The data that support the findings of this study are available from the corresponding author upon reasonable request.
