# Supplementary material for: Disgust Propensity, Not Disgust Sensitivity, Shapes the Reactivity of a Subjective Disgust Circuit in Humans
Source: Hum Brain Mapp. 2026 Jun 4;47(8):e70577. doi: 10.1002/hbm.70577 (PMC13581101; doi:10.1002/hbm.70577)
Supplement: Supplementary file 1 [file HBM-47-e70577-s001.docx]

Disgust propensity, not disgust sensitivity, shapes the reactivity of a subjective disgust circuit in humans

Xianyang Gan^1,2^, Zihao Zheng^1,2^, Ran Zhang^3,4^, Feng Zhou^3,4^, Ting Xu^3,4^, Nan Qiu^1,2^, Junjie Wang^1,2^, Heng Jiang^1,2^, Shan Gao^5^, Yu Wu^6^, Benjamin Klugah-Brown^1,2^, Dezhong Yao^1,2,*^ and Benjamin Becker^6,7,*^

^1^The Clinical Hospital of Chengdu Brain Science Institute, MOE Key Lab for Neuroinformation, School of Life Science and Technology, University of Electronic Science and Technology of China, Chengdu, China

^2^China-Cuba Belt and Road Joint Laboratory on Neurotechnology and Brain-Apparatus Communication, University of Electronic Science and Technology of China, Chengdu, China

^3^Faculty of Psychology, Southwest University, Chongqing, China

^4^Key Laboratory of Cognition and Personality, Ministry of Education, Chongqing, China

^5^School of Foreign Languages, University of Electronic Science and Technology of China, Chengdu, China

^6^MIND & AI Lab, Department of Psychology, The University of Hong Kong, Hong Kong SAR, China

^7^SRT AI, Society & Social Dynamics, Faculty of Social Sciences, The University of Hong Kong, Hong Kong SAR, China

* Authors for correspondence:

Dezhong Yao

e-mail: dyao@uestc.edu.cn

Benjamin Becker

e-mail: ben_becker@gmx.de

**The Chinese version of DPSS-R**

(for the original English version, see van Overveld, de Jong, Peters, Cavanagh, & Davey, 2006)

**指导语：**该问卷包含了16个与厌恶/恶心情绪相关的题项。请仔细阅读每个题项，根据每个题项与你的符合程度进行相应的评分。评分范围从1到5，分别代表从“从不”到“总是”的不同程度，选择数字越大，表示符合程度越高。请认真回答每一道题，你只需要根据自己的第一感觉作答即可，不必过多思考。请在符合的地方打上勾“√”。

|  | **从不** | **很少** | **有时** | **经常** | **总是** |
| --- | --- | --- | --- | --- | --- |
| 1. 我会避免接触令人厌恶的事物。 |  |  |  |  |  |
| 2. 当我感到厌恶时，我担心自己会晕倒。 |  |  |  |  |  |
| 3. 当我感到厌恶时，我会惊慌失措。 |  |  |  |  |  |
| 4. 我觉得厌恶的事物可能会导致我生病/感染。 |  |  |  |  |  |
| 5. 我时常产生厌恶情绪。 |  |  |  |  |  |
| 6. 厌恶的事物让我反胃。 |  |  |  |  |  |
| 7. 我会将厌恶情绪表现在脸上。 |  |  |  |  |  |
| 8. 一旦察觉到厌恶情绪，我担心自己会吐出来。 |  |  |  |  |  |
| 9. 每当厌恶情绪来袭，我会有强烈的感受。 |  |  |  |  |  |
| 10. 我时常感到厌恶。 |  |  |  |  |  |
| 11. 当我感到头晕时，我会惊慌失措。 |  |  |  |  |  |
| 12. 我比别人更容易产生厌恶情绪。 |  |  |  |  |  |
| 13. 我时常担心自己可能会咽下一个恶心的食物。 |  |  |  |  |  |
| 14. 我厌恶某些特定的事物。 |  |  |  |  |  |
| 15. 当我感到厌恶时，它会使我感到尴尬。 |  |  |  |  |  |
| 16. 我认为厌恶情绪于我而言很糟糕。 |  |  |  |  |  |

评分说明：从不 = 1，很少 = 2，有时 = 3，经常 = 4，总是 = 5。

厌恶倾向性得分：第 1, 5, 6, 7, 9, 10, 12, 14 题的总和。

厌恶敏感性得分：第 2, 3, 4, 8, 11, 13, 15, 16 题的总和。

**Supplementary Methods**

**MRI data acquisition**

The MRI data of the first experiment were acquired on a GE MR750 (General Electric Medical System) 3T MRI system. Functional images were obtained with a T2*-weighted echo planar imaging (EPI) sequence with the following parameters: repetition time = 2000  ms, echo time = 30  ms, flip angle = 90°, field of view = 240 × 240  mm, voxel size = 3.75 × 3.75 × 4  mm, resolution = 64 × 64, number of slices = 39, slice thickness = 3  mm, and an inter-slice gap of 1 mm. High-resolution structural images were acquired using a T1-weighted spoiled gradient recall (SPGR) sequence with the following parameters: repetition time = 6 ms, echo time = 2 ms, flip angle = 9°, field of view = 256 × 256 mm, voxel size = 1 × 1 × 1 mm, acquisition matrix = 256 × 256, number of slices = 156, and slice thickness = 1 mm. These structural images were utilized to enhance spatial normalization and to exclude participants with evident brain pathologies.

The MRI data of the second experiment were acquired on a GE MR750 3T MRI system. Functional images were obtained with a T2*-weighted EPI sequence with the following parameters: repetition time = 2000  ms, echo time = 30  ms, flip angle = 90°, field of view = 200 × 200  mm, voxel size = 3.125×3.125×3.8  mm, resolution = 64 × 64, number of slices = 36, slice thickness = 3.8  mm, and no gap. High-resolution structural images were acquired using a T1-weighted SPGR sequence with the following parameters: repetition time = 8 ms, echo time = 3 ms, flip angle = 8°, field of view = 256 × 256 mm, voxel size = 1 × 1 × 1 mm, acquisition matrix = 256 × 256, number of slices = 176, and slice thickness = 1 mm. These structural images were used to improve spatial normalization and to exclude participants with apparent brain pathologies.

**Modeling trial-wise subjective disgust using parametric modulation**

We employed a parametric modulation approach to model trial-wise variation in subjective disgust experience. At the first level, two regressors of interest were specified: one modeling the picture presentation period and one modeling the rating period. Subjective disgust ratings (1–5) for each trial were included as a parametric modulator of the picture presentation regressor. The model also included one boxcar regressor indicating the 4 s rating period to account for motor-related activity. Task regressors were convolved with the canonical hemodynamic response function, and a high-pass filter of 128 s was applied. The fMRI task runs were concatenated for each participant (four runs for the first experiment and two runs for the second experiment) using the SPM function spm_fmri_concatenate.m. Nuisance regressors included: (1) run-specific intercepts; (2) 24 motion parameters (six realignment parameters, their squares, derivatives, and squared derivatives); and (3) regressors modeling motion outlier timepoints.

At the second level, we performed regression analyses to examine whether inter-individual variability in parametric modulation effects (i.e., trial-wise coupling between subjective ratings and neural responses) was associated with disgust propensity and disgust sensitivity, respectively. Given that no effects survived cluster-level FWE correction, results are reported at a voxel-level threshold of *P* < 0.001 (uncorrected, k > 30) and should be considered exploratory.

**Activation likelihood estimation (ALE)**

The ALE method estimates the probability of each voxel being activated across multiple independent experiments, taking into account the number of participants in each study. In other words, ALE seeks to determine whether the convergence of activation foci across included experiments occurs at a level greater than chance (Laird et al., 2005). Specifically, ALE treats the reported peak foci from single studies as three-dimensional Gaussian probability distributions and provides empirical estimates to address the spatial uncertainty that may arise due to between-subject and between-template variability of the neuroimaging foci (Eickhoff et al., 2009). For each Gaussian probability distribution function, the full-width at half-maximum is weighted by the sample size of the respective experiment, such that experiments with larger samples produce smaller Gaussian distributions, reflecting more precise and reliable estimates of “true” activation (Eickhoff et al., 2009).

For each included study, modeled activation maps were generated by combining the probabilities of all activation foci for each voxel (Eickhoff et al., 2009; Turkeltaub et al., 2012). The union of individual modeled activation maps across experiments was calculated to produce an ALE map, reflecting brain regions (clusters) where activation convergence was observed (Eickhoff, Bzdok, Laird, Kurth, & Fox, 2012; Eickhoff et al., 2009). Consistent with standard practices, the ALE map was thresholded using a cluster-level familywise error (cluster-FWE) correction at *P* < 0.05, with an initial cluster-forming threshold set at the voxel-level of *P* < 0.001 (uncorrected) (Eickhoff, Laird, Fox, Lancaster, & Fox, 2017; Eickhoff et al., 2016; Ferraro et al., 2022; Gan et al., 2022; Klugah-Brown et al., 2020; Klugah-Brown et al., 2021). The *P* value reflects the proportion of the null distribution of random spatial relation across experiments. In our analyses, *P* values were generated by 5000 permutations (Gan et al., 2022; Laird et al., 2010; Tao, He, Lin, Liu, & Tao, 2021).

**Data acquisition parameters and preprocessing procedures of the OASIS-3 dataset**

Images were acquired on a Siemens TimTrio 3T scanner. Resting-state functional images were obtained using an echo-planner imaging sequence using the following parameters: repetition time = 2200 ms, echo time = 27 ms, flip angle = 90°, voxel size = 4.0 × 4.0 × 4.0 mm^3^, 33 slices, and slice thickness  =  4 mm. Preprocessing was conducted using Data Processing Assistant for Resting-State fMRI (DPARSF, v.5.0_201001) (http://rfmri.org/DPARSF). In brief, the preprocessing pipeline included the following steps: (1) discarding the first five volumes to improve stability of the magnetic field, (2) slice-timing correction to account for temporal differences in slice acquisition, (3) realignment to correct for head motion, (4) normalization of functional images to the standard MNI space, (5) grey matter, white matter, cerebrospinal fluid, and head motion regression, (6) detrending, (7) filtering (0.01 - 0.1 Hz), and (8) spatial smoothing with a 6-mm full-width at half-maximum Gaussian kernel.

**Supplementary Results**

**Correlation results for reaction time and trait disgust measures**

To examine whether individual differences in reaction time were related to trait disgust measures, we calculated correlations between reaction time indices and both disgust propensity and disgust sensitivity. Overall, none of the correlations reached statistical significance (all *P*s > 0.10).

Specifically, the correlation between the mean reaction time for high disgust pictures and disgust propensity was 0.03 (*P* = 0.72), while the correlations between disgust propensity and the mean reaction time for moderate disgust and neutral pictures were -0.10 (*P* = 0.25) and -0.001 (*P* = 0.99), respectively. In addition, the correlations between disgust propensity and reaction time difference scores were also non-significant, including the difference between high disgust and neutral pictures (*r* = -0.03, *P* = 0.69), high disgust and moderate disgust pictures (*r* = -0.10, *P* = 0.24), and moderate disgust and neutral pictures (*r* = -0.06, *P* = 0.45).

Similarly, none of the correlations between reaction time measures and disgust sensitivity reached statistical significance. The correlation between the mean reaction time for high disgust pictures and disgust sensitivity was −0.06 (*P* = 0.51), while the correlations for moderate disgust and neutral pictures were −0.13 (*P* = 0.11) and −0.06 (*P* = 0.49), respectively. The reaction time difference scores were also not significantly correlated with disgust sensitivity, including the difference between high disgust and neutral pictures (*r* = 0.03, *P* = 0.72), high disgust and moderate disgust pictures (*r* = −0.04, *P* = 0.66), and moderate disgust and neutral pictures (*r* = 0.01, *P* = 0.87).

**Regression results for the condition of high disgust > moderate disgust**

Applying the same threshold (i.e., cluster-level FWE *P* < 0.05 correction and voxel-level *P* < 0.001) to the condition of high disgust > moderate disgust did not yield any results. Given this, we reported the regression results at the voxel-level *P* < 0.001 to observe some trend. The results showed that activation to high disgust stimuli (high disgust > moderate disgust) was significantly modulated by participants’ disgust propensity scores within the bilateral anterior, mid- and posterior insular cortex, bilateral occipital visual cortex, right putamen, left thalamus, left hippocampus, left parahippocampal gyrus, left caudate, and left inferior/middle frontal gyrus (Table S2 and Fig. S2).

**Regression results for the condition of moderate disgust > neutral**

Applying the same threshold (i.e., cluster-level FWE *P* < 0.05 correction and voxel-level *P* < 0.001) to the condition of moderate disgust > neutral failed to yield any results. We thus relax the threshold to voxel-level *P* < 0.001. The results showed that activation to moderate disgust stimuli (moderate disgust > neutral) was significantly modulated by participants’ disgust propensity scores in the right precentral gyrus, right postcentral gyrus, bilateral paracentral lobule, and right middle/superior frontal gyrus (Table S3 and Fig. S3).

Table S1. Brain areas where activation to viewing high disgust stimuli (high disgust > neutral) was signiﬁcantly associated with participants' scores on the disgust propensity subscale (further including sex and the reaction time difference for high disgust versus neutral pictures as covariates in the regression analysis)

| Regions | Side | MNI peak coordinates  (x, y, z) | | | *t*-value | Cluster Size | Cluster-FWE |
| --- | --- | --- | --- | --- | --- | --- | --- |
| Anterior Insula, Middle Insula, Posterior Insula, Caudate, Putamen | R | 34 | 4 | 14 | 4.52 | 511 | 0.012 |
| Thalamus, Hippocampus, Caudate, Parahippocampal Gyrus | L | -24 | -32 | 10 | 4.65 | 450 | 0.019 |

Note: voxel-level uncorrected *P* < 0.001, combined with cluster-level FWE correction. Abbreviations: L, left; R, right.

Table S2. Brain areas where activation to viewing high disgust stimuli (high disgust > moderate disgust) was signiﬁcantly associated with participants' scores on the disgust propensity subscale

| Regions | Side | MNI peak coordinates  (x, y, z) | | | *t*-value | Cluster Size |
| --- | --- | --- | --- | --- | --- | --- |
| Anterior Insula, Middle Insula, Posterior Insula, Putamen | R | 34 | 4 | 14 | 5.67 | 222 |
| Thalamus | L | -14 | -26 | 14 | 4.44 | 112 |
| Hippocampus, Parahippocampal Gyrus, Caudate | L | -34 | -32 | -2 | 4.18 | 144 |
| Inferior Occipital Gyrus, Lingual Gyrus, Middle Occipital Gyrus | L | -18 | -96 | -6 | 4.00 | 151 |
| Anterior Insula, Middle Insula, Posterior Insula | L | -36 | 0 | 14 | 3.84 | 51 |
| Middle Frontal Gyrus, Inferior Frontal Gyrus | L | -32 | 32 | 16 | 3.72 | 57 |
| Anterior Insula, Middle Insula | L | -30 | 12 | 14 | 3.70 | 37 |
| Lingual Gyrus, Inferior Occipital Gyrus, Middle Occipital Gyrus | R | 20 | -96 | -4 | 3.51 | 118 |

Note: voxel-level uncorrected *P* < 0.001. Abbreviations: L, left; R, right.

Table S3. Brain areas where activation to viewing moderate disgust stimuli (moderate disgust > neutral) was signiﬁcantly associated with participants' scores on the disgust propensity subscale

| Regions | Side | MNI peak coordinates  (x, y, z) | | | *t*-value | Cluster Size |
| --- | --- | --- | --- | --- | --- | --- |
| Precentral Gyrus | R | 46 | 0 | 32 | 3.88 | 55 |
| Precentral Gyrus, Postcentral Gyrus | R | 44 | -18 | 68 | 3.78 | 45 |
| Superior Frontal Gyrus, Middle Frontal Gyrus | R | 26 | 58 | -20 | 3.77 | 61 |
| Paracentral Lobule | R/L | 4 | -30 | 60 | 3.62 | 68 |

Note: voxel-level uncorrected *P* < 0.001. Abbreviations: L, left; R, right.

Table S4. Brain regions where parametric modulation of neural responses by subjective disgust ratings was significantly associated with participants' scores on the disgust propensity subscale

| Regions | Side | MNI peak coordinates  (x, y, z) | | | *t*-value | Cluster Size |
| --- | --- | --- | --- | --- | --- | --- |
| Caudate | R | 12 | 0 | 26 | 4.18 | 161 |
| Thalamus, Hippocampus, Caudate | L | -24 | -36 | 10 | 4.08 | 136 |
| Parahippocampal Gyrus, Fusiform Gyrus, Middle Temporal Gyrus | L | -38 | -50 | -6 | 4.08 | 108 |
| Thalamus, Hippocampus, Caudate | R | 20 | -34 | 10 | 4.15 | 102 |
| Superior Temporal Gyrus, Middle Temporal Gyrus | R | 70 | -48 | 12 | 3.90 | 105 |
| Superior Temporal Gyrus, Middle Temporal Gyrus | R | 42 | -50 | 10 | 3.56 | 89 |
| Postcentral Gyrus, Precentral Gyrus | R | 24 | -36 | 50 | 3.99 | 84 |
| Anterior Insula, Middle Insula, Posterior Insula | R | 34 | 4 | 14 | 3.98 | 79 |
| Middle Temporal Gyrus, Superior Temporal Gyrus | R | 46 | -26 | -6 | 3.75 | 61 |
| Hippocampus, Caudate | L | -32 | -32 | 0 | 4.31 | 47 |
| Caudate | L | -12 | -2 | 24 | 3.80 | 44 |
| Inferior Parietal Lobule, Supramarginal Gyrus | R | 62 | -28 | 26 | 3.43 | 41 |

Note: voxel-level uncorrected *P* < 0.001. Abbreviations: L, left; R, right.

Table S5. Conjunction analysis of MACM and RSFC results for the right anterior insula from regression analysis

| Regions | Side | MNI peak coordinates  (x, y, z) | | | Cluster Size |
| --- | --- | --- | --- | --- | --- |
| Anterior Insula, Middle Insula, Posterior Insula, Putamen, Caudate, Thalamus, Precentral Gyrus | R | 34 | 4 | 12 | 1394 |
| Thalamus, Anterior Insula, Middle Insula, Posterior Insula, Putamen, Caudate, Precentral Gyrus | L | -14 | -16 | 6 | 775 |
| Inferior Parietal Lobule, Supramarginal Gyrus, Postcentral Gyrus | R | 48 | -34 | 26 | 233 |
| Inferior Parietal Lobule | L | -54 | -30 | 24 | 13 |
| Midcingulate Cortex, Supplementary Motor Area | R/L | 8 | 12 | 38 | 297 |

Abbreviations: L, left; R, right.

Table S6. Conjunction analysis of MACM and RSFC results for the left thalamus from regression analysis

| Regions | Side | MNI peak coordinates  (x, y, z) | | | Cluster Size |
| --- | --- | --- | --- | --- | --- |
| Thalamus, Hippocampus, Posterior Insula, Caudate, Parahippocampal Gyrus | L | -22 | -32 | 8 | 384 |
| Thalamus, Hippocampus, Parahippocampal Gyrus | R | 28 | -32 | 2 | 56 |

Abbreviations: L, left; R, right.

Fig. S1 Positive association between disgust propensity scores and brain activation while viewing high disgust stimuli (high disgust > neutral), with sex and the reaction time difference for high disgust versus neutral pictures as covariates. Note: activated voxel group shown at a statistical threshold of voxel-level *P* < 0.001 and cluster-level *P* < 0.05. Abbreviations: aIns, anterior insula; Cau, caudate; Hipp, hippocampus; mIns, middle insula; Parahipp, parahippocampal gyrus; pIns, posterior insula; Put, putamen; Thal, thalamus.

Fig. S2 Positive association between disgust propensity scores and brain activation while viewing high disgust stimuli (high disgust > moderate disgust). Note: activated voxel group shown at a statistical threshold of voxel-level *P* < 0.001. Abbreviations: aIns, anterior insula; Cau, caudate; Hipp, hippocampus; IFG, inferior frontal gyrus; IOG, inferior occipital gyrus; LG, lingual gyrus; MFG, middle frontal gyrus; mIns, middle insula; MOG, middle occipital gyrus; Parahipp, parahippocampal gyrus; pIns, posterior insula; Put, putamen; Thal, thalamus.

Fig. S3 Positive association between disgust propensity scores and brain activation while viewing moderate disgust stimuli (moderate disgust > neutral). Note: activated voxel group shown at a statistical threshold of voxel-level *P* < 0.001. Abbreviations: MFG, middle frontal gyrus; PL, paracentral lobule; PostG, postcentral gyrus; PreG, precentral gyrus; SPG, superior frontal gyrus.

Fig. S4 Positive association between disgust propensity and parametric modulation of neural responses by subjective disgust ratings. Note: activated voxel group shown at a statistical threshold of voxel-level *P* < 0.001. Abbreviations: aIns, anterior insula; Cau, caudate; Hipp, hippocampus; IPL, inferior parietal lobule; mIns, middle insula; Parahipp, parahippocampal gyrus; pIns, posterior insula; PostG, postcentral gyrus; STG, superior temporal gyrus; Thal, thalamus.

Fig. S5 Mediation analyses exploring whether trait disgust mediates the relationship between state disgust and the mean activation (from regression results that included sex and the reaction time difference for high disgust versus neutral pictures as covariates) in the anterior insula and thalamus clusters. (a) Disgust propensity fully mediates the association between state disgust and the mean activation of the anterior insula cluster. (b) Disgust sensitivity does not mediate the effect of state disgust on the mean activation of the anterior insula cluster. (c) Disgust propensity fully mediates the association between state disgust and the mean activation of the thalamus cluster. (d) Disgust sensitivity fails to mediate the effect of state disgust on the mean activation of the thalamus cluster. ****P* < 0.001, NS not significant.

Fig. S6 Connectivity profiles of the right anterior insula from regression analysis. (a) Task-based connectivity map (i.e., MACM). (b) Task-free connectivity map (i.e., RSFC). (c) Consensus connectivity map (i.e., MACM ∩ RSFC, where ∩ indicates conjunction). Abbreviation: ALE, activation likelihood estimation.

Fig. S7 Connectivity profiles of the left thalamus from regression analysis. (a) Task-based connectivity map (i.e., MACM). (b) Task-free connectivity map (i.e., RSFC). (c) Consensus connectivity map (i.e., MACM ∩ RSFC, where ∩ indicates conjunction). Abbreviation: ALE, activation likelihood estimation.

**References**

Eickhoff, S. B., Bzdok, D., Laird, A. R., Kurth, F., & Fox, P. T. (2012). Activation likelihood estimation meta-analysis revisited. *Neuroimage, 59*(3), 2349-2361. <https://doi.org/10.1016/j.neuroimage.2011.09.017>

Eickhoff, S. B., Laird, A. R., Fox, P. M., Lancaster, J. L., & Fox, P. T. (2017). Implementation errors in the gingerale software: Description and recommendations. *Human Brain Mapping, 38*(1), 7-11. <https://doi.org/10.1002/hbm.23342>

Eickhoff, S. B., Laird, A. R., Grefkes, C., Wang, L. E., Zilles, K., & Fox, P. T. (2009). Coordinate-based activation likelihood estimation meta-analysis of neuroimaging data: A random-effects approach based on empirical estimates of spatial uncertainty. *Human Brain Mapping, 30*(9), 2907-2926. <https://doi.org/10.1002/hbm.20718>

Eickhoff, S. B., Nichols, T. E., Laird, A. R., Hoffstaedter, F., Amunts, K., Fox, P. T., . . . Eickhoff, C. R. (2016). Behavior, sensitivity, and power of activation likelihood estimation characterized by massive empirical simulation. *Neuroimage, 137*, 70-85. <https://doi.org/10.1016/j.neuroimage.2016.04.072>

Ferraro, S., Klugah-Brown, B., Tench, C. R., Bazinet, V., Bore, M. C., Nigri, A., . . . Becker, B. (2022). The central autonomic system revisited - convergent evidence for a regulatory role of the insular and midcingulate cortex from neuroimaging meta-analyses. *Neuroscience & Biobehavioral Reviews, 142*, 104915. <https://doi.org/10.1016/j.neubiorev.2022.104915>

Gan, X., Zhou, X., Li, J., Jiao, G., Jiang, X., Biswal, B., . . . Becker, B. (2022). Common and distinct neurofunctional representations of core and social disgust in the brain: Coordinate-based and network meta-analyses. *Neuroscience & Biobehavioral Reviews, 135*, 104553. <https://doi.org/10.1016/j.neubiorev.2022.104553>

Klugah-Brown, B., Di, X., Zweerings, J., Mathiak, K., Becker, B., & Biswal, B. (2020). Common and separable neural alterations in substance use disorders: A coordinate-based meta-analyses of functional neuroimaging studies in humans. *Human Brain Mapping, 41*(16), 4459-4477. <https://doi.org/10.1002/hbm.25085>

Klugah-Brown, B., Zhou, X., Pradhan, B. K., Zweerings, J., Mathiak, K., Biswal, B., & Becker, B. (2021). Common neurofunctional dysregulations characterize obsessive-compulsive, substance use, and gaming disorders - an activation likelihood meta-analysis of functional imaging studies. *Addiction Biology, 26*(4), e12997. <https://doi.org/10.1111/adb.12997>

Laird, A. R., Fox, P. M., Price, C. J., Glahn, D. C., Uecker, A. M., Lancaster, J. L., . . . Fox, P. T. (2005). Ale meta-analysis: Controlling the false discovery rate and performing statistical contrasts. *Human Brain Mapping, 25*(1), 155-164. <https://doi.org/10.1002/hbm.20136>

Laird, A. R., Robinson, J. L., McMillan, K. M., Tordesillas-Gutiérrez, D., Moran, S. T., Gonzales, S. M., . . . Lancaster, J. L. (2010). Comparison of the disparity between talairach and mni coordinates in functional neuroimaging data: Validation of the lancaster transform. *Neuroimage, 51*(2), 677-683. <https://doi.org/10.1016/j.neuroimage.2010.02.048>

Tao, D., He, Z., Lin, Y., Liu, C., & Tao, Q. (2021). Where does fear originate in the brain? A coordinate-based meta-analysis of explicit and implicit fear processing. *Neuroimage, 227*, 117686. <https://doi.org/10.1016/j.neuroimage.2020.117686>

Turkeltaub, P. E., Eickhoff, S. B., Laird, A. R., Fox, M., Wiener, M., & Fox, P. (2012). Minimizing within-experiment and within-group effects in activation likelihood estimation meta-analyses. *Human Brain Mapping, 33*(1), 1-13. <https://doi.org/10.1002/hbm.21186>

van Overveld, M., de Jong, P. J., Peters, M. L., Cavanagh, K., & Davey, G. C. L. (2006). Disgust propensity and disgust sensitivity: Separate constructs that are differentially related to specific fears. *Personality and Individual Differences, 41*(7), 1241-1252. <https://doi.org/10.1016/j.paid.2006.04.021>
